# A Positive Association of Overactivated Immunity with Metabolic Syndrome Risk and Mitigation of Its Association by a Plant-Based Diet and Physical Activity in a Large Cohort Study

**DOI:** 10.3390/nu13072308

**Published:** 2021-07-05

**Authors:** Sunmin Park, Ting Zhang

**Affiliations:** Food and Nutrition, Obesity/Diabetes Research Center, Hoseo University, 165 Sechul-Ri, Asan-si 336-795, ChungNam-do, Korea; zhangting92925@gmail.com

**Keywords:** white blood cell count, plant-based diet pattern, Western-style diet pattern, serum CRP concentrations, type 2 diabetes, metabolic syndrome

## Abstract

The association between immunity and metabolic syndrome (MetS) has been studied, but its interaction with lifestyles remains unclear. We studied their association and interactions with lifestyles in 40,768 adults aged over 40 years from a large-scale, hospital-based cohort study collected during 2010–2013. White blood cell counts (WBC) and serum C-reactive protein concentrations (CRP) were used as indexes of immune status. The participants were categorized into four groups by the cutoff points of 6.2 × 10^9^/L WBC(L-WBC) and <0.5 mg/dL CRP(L-CRP): L-WBC+L-CRP(*n* = 25,604), H-WBC+L-CRP(*n* = 13,880), L-WBC+H-CRP(*n* = 464), and H-WBC+H-CRP(*n* = 820). The participants in the H-WBC+L-CRP were younger and had higher numbers of males than the L-WBC+L-CRP. MetS risk was higher by 1.75- and 1.86-fold in the H-WBC+L-CRP and H-WBC+H-CRP, respectively, than the L-WBC+L-CRP. MetS components, including plasma glucose and triglyceride concentrations, and SBP were elevated in H-WBC+L-CRP and H-WBC+H-CRP compared with L-WBC+L-CR+P. The risk of hyperglycemia and high HbA1c was the highest in the H-WBC+H-CRP among all groups. Areas of WBC counts and serum CRP concentrations were 0.637 and 0.672, respectively, in the receiver operating characteristic curve. Daily intake of energy, carbohydrate, protein, and fat was not significantly different in the groups based on WBC counts and CRP. However, a plant-based diet (PBD), physical activity, and non-smoking were related to lowering WBC counts and CRP, but a Western-style diet was linked to elevating CRP. A high PBD intake and smoking status interacted with immunity to influence MetS risk: a low PBD and current smoking were associated with a higher MetS risk in the H-WBC+H-CRP. In conclusion, overactivated immunity determined by CRP and WBC was associated with MetS risk. Behavior modification with PBD and physical activity might be related to immunity regulation.

## 1. Introduction

In the pandemic era, maintaining optimal immunity plays a crucial role in protecting against pathogens. Immunity is achieved with innate and adaptive immune responses. The innate immune system is activated to nonspecifically eliminate pathogens as the first defense line when pathogens attack the body [1]. The innate immune system acts as the connector to adaptive immunity by producing specific antigens from the infected pathogen by lymphocytes, which subsequently initiates adaptive immunity [1]. The innate immune response subsequently subsides with inflammation, which returns to the normal state [1]. Chronic metabolic disorders, including type 2 diabetes and obesity, contribute to the chronic activation of innate immune responses that impairs the increase of adaptive immunity when an infection occurs [2]. In developing countries, impaired immune function is also associated with the onset of sepsis and infectious diseases. In modern, industrialized societies, people have chronic, low-grade inflammation due to immune overactivation. It is associated with worsening cardiovascular diseases and autoimmune diseases [3]. Therefore, immune overactivation by overnutrition, sedentary lifestyle, and metabolic disorders, such as metabolic syndrome (MetS) and cardiovascular diseases [3].

However, individuals’ immune status is difficult to measure in a clinical setting since optimal markers for immune function are not clear. White blood cells (WBC; leukocytes) eliminate foreign particles, pathogens, and cell debris. WBC includes natural killer cells, mast cells, eosinophils, basophils, macrophages, neutrophils, and dendritic cells whose combined actions form the innate immune response [4,5]. Furthermore, WBC is also associated with adaptive immunity [6]. WBC is an indicator of immune status to recruit participants with an immune deficiency in intervention studies [7,8]. Proinflammatory cytokines result in excessive immune activity and vice versa [9]. The increase of serum IL-6 concentrations produced by various cells, including macrophage and adipose tissues, elevates C-reactive protein (CRP) secretion related to the overactivation of immunity [10,11]. Therefore, increased WBC counts and serum CRP concentrations indicate overactivated immunity. Since it is challenging to assess immune status in large populations, combining the WBC counts and serum CRP concentrations can represent immune status. 

Immune status is associated with lifestyles, especially nutrient intake and dietary patterns. An unhealthy diet, mental stress, cigarette smoking, and sedentary life may increase chronic inflammation, an abnormal immune response [5]. The gut microbiome also influences systemic inflammation, and probiotics and dietary fiber modify the gut microbiome. Sufficient intake of dietary fiber improves increased immunity and inflammation [12]. By contrast, high saturated fat diets increase pathogens in the gut, and they damage the intestinal epithelial cell layer to increase inflammation, possibly through overactivation of immunity [13]. Therefore, lifestyles are involved in modulating immunity and inflammation status.

Here, we hypothesized that overactivated immunity was associated with MetS risk, and they had interactions with lifestyles, including dietary patterns. The hypothesis was examined in 40,768 adults aged over 40 years in a large city hospital cohort study. This study supported that overactive immunity might be associated with MetS risk, and lifestyle changes have the potential to suppress it, leading to being positively associated with MetS risk. 

## 2. Materials and Methods

### 2.1. Subjects

The 58,701 participants, ≥40 years old, were recruited from a large urban hospital cohort study in the Korean Genome and Epidemiology Study (KoGES) conducted during 2010–2014 [14]. Participants in whom WBC counts were not measured were removed (*n* = 17,933). The 40,768 remaining participants were included in the analysis. The institutional review board (IRB) of the Korean National Institute of Health approved the KoGES (KBP-2015-055), and the IRB of Hoseo University (1041231-150811-HR-034-01) approved the present study. Written informed consent was received from all participants. 

### 2.2. Basal Characteristics and Anthropometric and Biochemical Parameters of the Participants

All participants had resided in their respective survey areas for at least six months. Information on age, education, income, smoking history, alcohol consumption, and physical activity was documented during a health interview. Smoking status was divided into three categories: current smoker (smoked at least 20 cigarettes in the past six months), past smoker, and never-smoker [15]. According to average daily alcohol consumption, alcohol consumption status was categorized into four groups (Table 1) [15]. Coffee intake was assessed in the same way as was alcohol intake. Regular physical activity was assessed by asking if subjects engaged in 30 min or more of physical activity for three or more days per week.

Anthropometric characteristics (e.g., height, weight, and body composition) were obtained at the initial interview as previously described [16,17] and were not part of the inclusion criteria. Obesity was defined as body mass index (BMI) ≥ 25 kg/m^2^ that was calculated by body weight (kg) divided by (height, m)^2^. Blood pressure was measured by a doctor via a sphygmomanometer under resting conditions three times, and the average systolic blood pressure (SBP) and diastolic blood pressure (DBP) were used to estimate hypertension. Blood was collected in heparin-treated tubes after fasting for >12 h, and plasma concentrations of total cholesterol, high-density lipoprotein cholesterol (HDL-C), and triglyceride and serum concentrations of glucose, alanine aminotransferase (ALT), aspartate aminotransferase (AST), and creatinine were measured using a Hitachi 7600 Automatic Analyzer (Hitachi, Tokyo, Japan). Serum CRP concentrations were measured using an ELISA kit. Blood HbA1c was measured using an automatic analyzer (ZEUS 9.9; Takeda, Tokyo, Japan). WBCs were counted from EDTA-treated blood. 

### 2.3. Definition of Immunity Status, MetS, and Immune-Related Diseases

Immunity status was categorized into four groups according to the WBC counts and serum CRP concentrations. Their cutoffs were <6.2 × 10^9^/L for WBC counts (L-WBC) and <0.5 mg/dL serum CRP concentrations (L-CRP) for low levels. Their high levels were indicated as H-WBC and H-CRP. Although 6.2 × 10^9^/L of WBC is within a normal range, the level was used for the cutoff value since it was the cutoff for metabolic syndrome incidence. The participants were categorized into L-WBC+L-CRP (*n* = 25,604), H-WBC+L-CRP(*n* = 13,880), L-WBC+H-CRP (*n* = 464), and H-WBC+H-CRP (*n* = 820). 

MetS was defined according to the 2005 revised National Cholesterol Education Program—Adult Treatment Panel III criteria for Asia [18,19]. Participants having three or more of the following criteria had MetS [18,19]: (1) elevated blood pressure (average systolic blood pressure ≥ 130 mmHg or diastolic blood pressure ≥ 85 mmHg) or current blood pressure medication use, (2) low HDL-C level (<40 mg/dL for men and <50 mg/dL for women), (3) elevated serum triglyceride level (≥150 mmol/L) or current anti-dyslipidemic medication use, (4) elevated fasting blood glucose level (≥100 mmol/L) or current use of anti-diabetic medication, and (5) abdominal obesity (waist circumference ≥ 90 cm for men and ≥85 cm for women).

Participants were asked whether they had a diagnosis of immunity-related diseases or cardiovascular diseases, including myocardial infarction and stroke. Medical histories of asthma, allergy, gastritis, bronchitis, and arthritis were questioned about immune-related diseases. Those who answered with yes for the disease diagnosis were considered to have the indicated diseases. 

### 2.4. Usual Food-Intake Measurement by a Semi-Quantitative Food Frequency Questionnaire (SQFFQ) 

Usual food intakes over the past six months were estimated using an SQFFQ in each of the participants. The accuracy and reproducibility of the SQFFQ for assessing the average consumption of food items during the previous year by Koreans have been validated [20]. The SQFFQ includes 103 food items that Koreans consume, and food frequencies were divided into the following nine categories: never or seldom, once per month, two to three times monthly, once or twice weekly, three or four times weekly, five or six times weekly, daily, twice daily, and ≥3 times daily. Food intakes per meal were scored as more than, equal to, or less than the standard portion size visualized using photographs of foods in each food category. Participants selected the frequencies of the portion size selected by the participants, and daily food intakes were computed by multiplying the median of weekly consumed frequencies by portion sizes in grams for each food category. Energy, protein, carbohydrates, fat, vitamin, and mineral intakes were calculated using Can-Pro 2.0 (Korean Nutrition Society, Seoul, Korea) nutrient intake assessment software developed by the Korean Nutrition Society.

### 2.5. Dietary Patterns by Principal Components Analysis

For dietary pattern analysis, 103 food items in the SQFFQ were made into 30 predefined food groups, as previously reported [21]. Dietary patterns were established by using principal components analysis of the 30 food groups. We determined the number of factors to retain based on eigenvalues > 1.5 in the principal component analysis and interpretability to extract four major dietary patterns. The variance of each dietary pattern explained the percentage of the corresponding food intake in the participants [22]. The orthogonal rotation procedure (varimax) yielded four dietary patterns uncorrelated to each other. Foods with ≥0.40 factor-loading values were considered to have a predominant contribution to the specific pattern [23]. Four dietary patterns were selected from the principal component analysis (Supplemental Table S1). These patterns indicate the participant’s diet types as having a more Korean balanced diet (KBD), plant-based diet (PBD), Western-style diet (WSD), or rice-based diet (RBD). These diet patterns were used to assess their effects on innate immune status and metabolic syndrome. 

### 2.6. Statistical Analysis

The statistical analysis was performed using SAS (version 9.3; SAS Institute, Cary, NC, USA). A sample size of 36,590 was sufficient to achieve significance at α = 0.05 and β = 0.99 at an odds ratio of 1.05 in the logistic analysis using a G-power calculator (University of Kiel, Kiel, Germany). Descriptive statistics for categorical variables, such as gender and dietary habits, were obtained by determining frequency distributions, which were statistically analyzed according to the immunity groups of classification variables using a chi-square test. Descriptive statistics of continuous variables are provided as adjusted means with standard deviations after adjusting for covariates, and the statistical differences among the four immunity groups were compared using a one-way analysis of covariance (ANCOVA) [24]. 

The association of immune status on metabolic parameters was examined using logistic regression analysis with L-WBC+L-CRP as a reference group after adjusting covariates. The logistic regression analysis was conducted considering two adjusted models. The first model included adjustment for age, residence area, survey year, BMI, education, job, income, and energy intake. The second model included adjustments for covariates in model 1 and physical activity, smoking, alcohol and coffee intake, allergy and arthritis medicine intake (mainly glucocorticoids), and having diseases related to immunity, including asthma, arthritis, allergy, gastritis, gastric ulcer, duodenal ulcer, cholecystitis, bronchitis, and urinary tract infection. The odds ratios (ORs) and 95% confidence intervals (CI) of each biochemical parameter for the immunity groups were provided with the logistic regression analysis. One-way ANCOVA assessed the statistical differences of the adjusted means among the four groups after adjusting covariates. Bonferroni correction was applied in the one-way ANCOVA test of the variables related to the hypothesis. Multiple comparisons of the immunity groups were performed using Tukey’s test. 

The lifestyle-related parameters were categorized into higher or lower groups using the criteria defined by the 70th percentiles of each variable to determine the interaction between the immunity groups and lifestyle parameters. The interactions between the immunity groups and the lifestyle parameters, including dietary intake, smoking, and physical activity, were analyzed using a two-way ANCOVA that included the main effects of lifestyle-related parameters on immunity and inflammation and their interaction term after adjusting covariates. The ORs and 95% CI of immunity with lifestyle-related parameters were also calculated by logistic regression analysis in the high and low groups of lifestyle-related parameters. 

The receiver operating characteristic (ROC) curve illustrates the diagnostic ability of immunity and MetS risk using the proc logistic model [25]. Classification analysis was performed by calculating sensitivity and specificity and area under the curve (AUC) of the ROC after adjusting for age, gender, waist circumferences, serum triglyceride and HDL concentrations, and blood pressure. The criterion value of WBC counts and serum CRP concentrations with maximum sensitivity and specificity was selected for the cutoff points. Area under the ROC curve (AUC) was categorized into 0.9–1.0 (excellent), 0.8–0.9 (good), 0.7–0.8 (fair), 0.6–0.7 (poor), and 0.5–0.6 (fail) for diagnosis ability of the diagnosis biomarker [25]. AUC of the ROC curve determined the diagnostic ability of the biomarker for immunity. The cutoff, sensitivity, and specificity values for immunity, determined by WBC counts and serum CRP concentrations, were calculated by the Youden’s index. A value of *p* < 0.05 was taken to indicate statistical significance.

## 3. Results

### 3.1. General Characteristics of the Participants according to Immunity Groups

The general characteristics of subjects are summarized according to WBC counts and serum CRP concentrations in Table 1. The average age of the participants in the H-WBC+L-CRP group was significantly lower, and it was significantly higher in the L-WBC+H-CRP and H-WBC+H-CRP groups than the L-WBC+L-CRP group. More male subjects belonged to the H-WBC+L-CRP and H-WBC+H-CRP than the other groups, whereas female participants were more prevalent in the L-WBC+L-CRP and L-WBC+H-CRP groups. More participants who answered “yes” for regular physical activity belonged to the L-WBC+L-CRP and L-WBC+HCRP groups than those who answered “no” (Table 1). The frequencies of obesity, hypertension, and MetS were highest in the H-WBC+H-CRP and lowest in the L-WBC+L-CRP (Table 1). The frequencies of myocardial infarction among cardiovascular diseases were higher in the H-WBC regardless of serum CRP concentrations among all groups, whereas stroke was highest in the H-WBC+H-CRP (Table 1). The general characteristics of men and women are presented according to immunity and gender in supplemental Table S1. They showed similar patterns in men and women. However, there were no significant differences in frequencies of cardiovascular diseases among the different immunity groups, which may have been due to the small number of patients in each group.

### 3.2. Metabolic Parameters According to Immunity Groups

According to the four immune status groups, WBC counts were highest in the H-WBC+H-CRP among the groups, but WBC counts were not significantly different between L-WBC+L-CRP and L-WBC+H-CRP (Bonferroni correction *p* < 0.002). Serum CRP concentrations were higher with H-WBC+H-CRP and H-WBC+L-CRP than with L-WBC+LCRP and L-WBC+H-CRP (Table 2; Bonferroni correction *p* < 0.002). MetS components, including abdominal visceral fat, hyperglycemia, and dyslipidemia, were significantly different, according to the four immune status groups (Table 2; Bonferroni correction *p* < 0.002). BMI and waist circumferences were lowest in the L-WBC+L-CRP, and waist circumferences were highest in the H-WBC+H-CRP among the groups (Table 2). Serum glucose concentrations, blood HBA1c, plasma total cholesterol, LDL cholesterol, and triglyceride concentrations showed similar relationships to BMI to immune status (Table 2). They were lower in order of the H-WBC+H-CRP, H-WBC+L-CRP, L-WBC+H-CRP, and L-WBC+L-CRP (Table 2). SBP and DBP were also significantly different among the immunity groups (*p* < 0.01) but not with Bonferroni correction.

### 3.3. Association of the Risk of Various Metabolic Diseases with Immunity Status

The participants aged ≥ 55 years had an inverse association with H-WBC+L-CRP (OR = 0.90; 95% CI = 0.86–0.95) compared with those with L-WBC+L-CRP, but they had a positive association with L-WBC+H-CRP (OR = 1.53; 95% = 1.23–1.89) and H-WBC+H-CRP (OR = 1.21; 95% CI = 1.03–1.42; Table 3). Females had a negative association with H-WBC+L-CRP (OR = 0.74) and H-WBC+H-CRP (OR = 0.09) compared with L-WBC+L-CRP (Table 3). MetS risk was positively associated, 1.75- and 1.86-folds, respectively, with H-WBC+L-CRP and H-WBC+H-CRP compared with L-WBC+L-CRP (Table 3). Among MetS components, BMI, low plasma HDL concentrations, HbA1c, SBP, and DBP had a positive association with H-WBC+L-CRP and H-WBC+H-CRP compared with L-WBC+L-CRP (Table 3). Waist circumferences showed no significant association with immunity groups after adjusting for covariates, including BMI (Table 3). Physical activity was inversely associated with H-WBC+L-CRP (OR = 0.82, 95% CI = 0.78–0.85) and H-WBC+H-CRP (OR = 0.67, 95% CI = 0.57–0.78) compared to L-WBC+L-CRP (Table 3). Furthermore, the associations of WBC or serum CRP concentrations with metabolic syndrome and its components are analyzed in supplemental Table S2. Metabolic syndrome and its components except DBP were positively associated with WBC and metabolic syndrome, BMI, waist circumferences, and serum HDL and AST concentrations were positively related to serum CRP concentrations. However, serum glucose concentrations, lipid profiles except for serum HDL concentrations, SBP, and DBP did not associate with serum CRP concentrations (supplemental Table S2).

**Figure 1:**
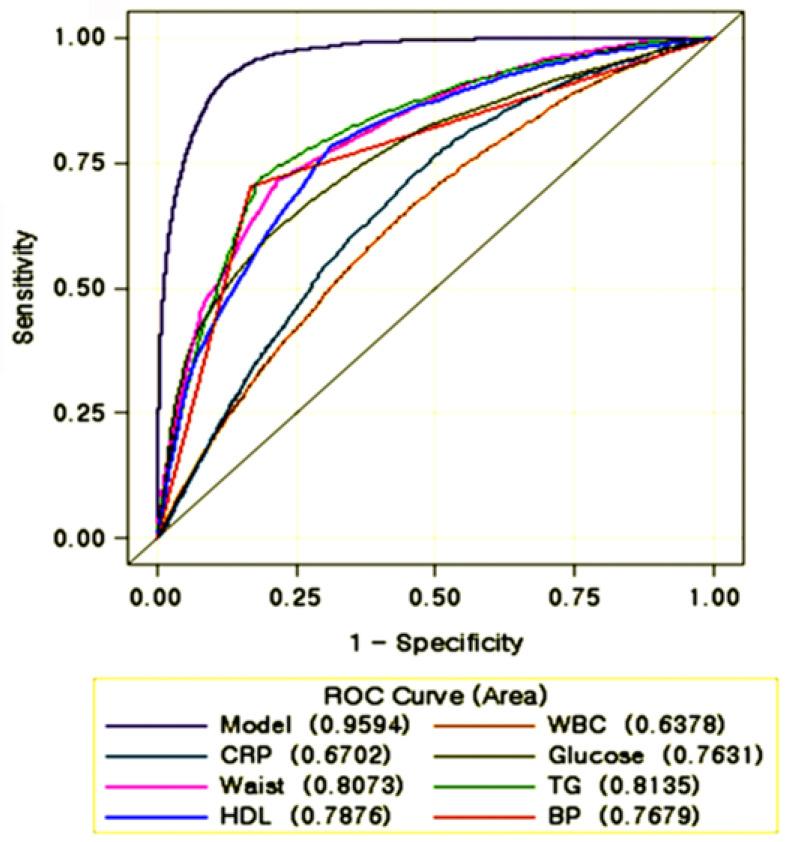
Receiver operating characteristic (ROC) curve with white blood cell (WBC) counts and serum C-reactive protein (CRP) concentrations for metabolic syndrome risk.

WBC counts and serum CRP concentrations were strongly associated with MetS risk by 1.31- and 1.39-folds, respectively (*p* < 0.001). Areas under the ROC curve (AUC) of WBC counts and serum CRP concentrations were 0.638 (95% CI = 0.629–0.647) and 0.670 (0.662–0.679) to estimate MetS risk, respectively, which illustrate a diagnostic ability (Figure 1). The 95 % CIs of the AUC for WBC count and serum CRP concentrations were above the reference value of 0.5 (Figure 1). These AUCs for immunity and inflammation were smaller than the MetS components, waist circumferences (0.807), serum concentrations of glucose (0.763), triglyceride (0.814), HDL (0.788), and blood pressure (0.768). The cutoff point of WBC was 6.220 × 10^9^/L in the highest Youden’s index = 0.5251, while the specificity and sensitivity of WBC counts were 0.7613 and 0.7613, respectively. The cutoff value of serum CRP concentrations was 0.494 mg/dL in the highest Youden’s index = 0.5251, while the specificity and sensitivity of serum CRP concentrations were 0.7613 and 0.7638, respectively. These results indicated that WBC counts and serum CRP concentrations alone are poor indicators for MetS, but their combination can be used as potential MetS risk indicators. The persons with WBC ≥ 6.2 × 10^9^/mL and serum CRP concentrations ≥ 0.49 mg/dL should be considered to have an elevated risk of MetS. 

### 3.4. Nutrient Intake Among the Immunity Groups

Energy intake was not significantly different among all groups. The carbohydrate intake was higher, and fat intake was lower in L-WBC+L-CRP than H-WBC+L-CRP. However, there was no significant difference in protein intake (Table 4). Vitamin intake as retinol and carotene and alcohol intakes were not significantly different among the immunity groups (Table 4). Vitamin C and dietary fiber intake were higher in L-WBC+H-CRP than H-WBC+L-CRP (*p* < 0.05). Interestingly, the participants with high coffee intake were higher in the H-WBC+L-CRP and H-WBC+H-CRP than L-WBC+L-CRP and L-WBC+H-CRP (*p* < 0.001). 

In PCA analysis, dietary patterns were categorized into four dietary patterns determined by eigenvector > 1.5: KBD, PBD, WSD, and RBD. The factor loading matrix showed which foods were included in each dietary pattern (Supplemental Table S3). The PCA revealed four dietary patterns, including KBD, PBD, WSD, and RBD, that explained 34.1, 30.6, 13.7, and 5.1% of the total variance in dietary intake, respectively. The four dietary patterns explained 83.5% of the total variance. Participants in the PBD group had higher scores in the food groups of beans, kimchi, mushroom, seaweeds, milk and milk products, cookies, fruits, and nuts (factor loading > 0.4), indicating higher adherence to the PBD pattern (Supplemental Table S3). Participants in the WSD were high in noodles, bread, red meats, soups, and chicken (factor loading > 0.4), suggesting higher adherence to the WSD pattern (Supplemental Table S2). For each individual, a pattern score was calculated. The scores indicate the participant’s adherence to a specific pattern, where a higher score indicates higher adherence to either of Korean balanced diet (KBD), plant-based diet (PBD), Western-style diet (WSD), or rice-based diet (RBD). The pattern scores were used to assess their effects on immune status and metabolic syndrome. Dietary patterns were associated with immunity groups (Table 4). However, there was no difference in KBD intake among the immunity groups. The PBD pattern was associated with L-WBC+L-CRP and was inversely associated with H-WBC+L-CRP and H-WBC+H-CRP (Table 4). By contrast, a high intake of WSD was inversely associated with L-WBC+L-CRP compared to H-WBC+L-CRP and H-WBC+H-CRP (Table 4). The RBD was also significantly less associated with L-WBC+L-CRP than the H-WBC+H-CRP.

### 3.5. Interactions of Nutrient Intake and Dietary Patterns with Immunity for Mets Risk 

**Figure 2:**
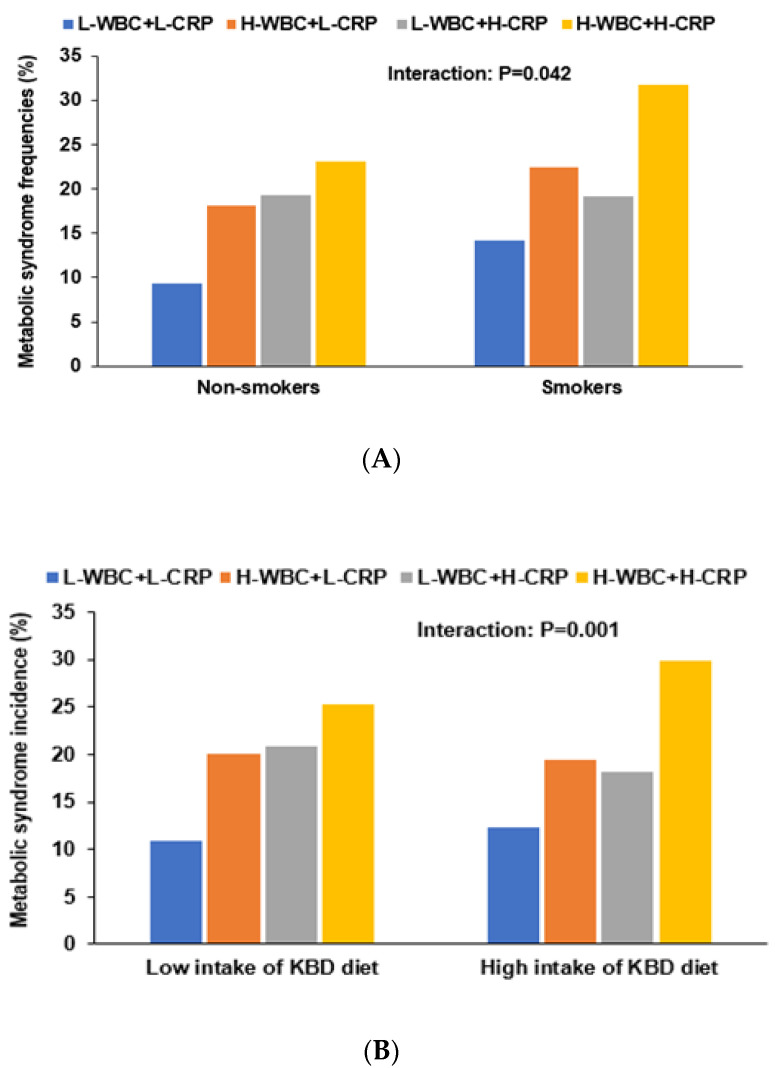

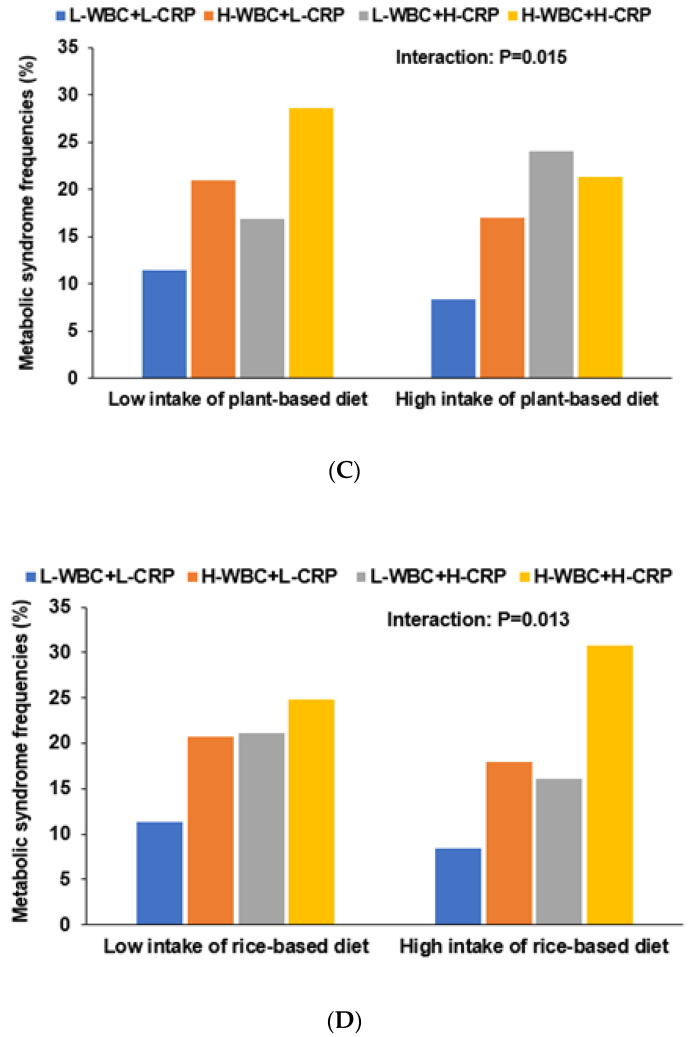
The frequency distribution of metabolic syndrome in four different groups categorized by white blood cell counts and serum CRP. (**A**) The incidence of metabolic syndrome of 4 groups according to smoking status. (**B**) The incidence of metabolic syndrome of 4 groups according to the intake of a Korean balanced diet pattern (cutoff point: 70th percentile). (**C**) The incidence of metabolic syndrome of 4 groups according to the intake of a plant-based diet pattern (cutoff point: 70th percentile). (**D**) The incidence of metabolic syndrome of 4 groups according to the intake of a rice-based diet pattern (cutoff point: 70th percentile).

Interaction between immunity and lifestyles, including diets, was assessed for MetS risk using two-way ANCOVA with main effects (immunity status and nutrients) and their interaction term after adjusting for covariates (Table 5). The participants with regular physical activity had lower immunity (Table 1), and physical activity was negatively associated with immunity, but they did not interact to influence MetS risk (*p* = 0.650; Table 5). The overall incidence of MetS was lower in the non-smokers compared to the smokers (Table 1). Current smoking had a positive association with immunity, and it had an interaction with immunity to influence MetS risk (*p* = 0.042; Table 5). In non-smokers, MetS risk was positively associated with the H-WBC+L-CRP group by 1.86-fold compared to the L-WBC+L-CRP, whereas in smokers, MetS risk was positively associated with the H-WBC+H-CRP group by 2.35-fold compared to the L-WBC+L-CRP (Table 5). The incidence of MetS was much higher in the H-WBC+H-CRP than the L-WBC+L-CRP in smokers (Figure 2A). 

Energy, protein, fat, carbohydrate, vitamin C, coffee and alcohol intake, and WSD pattern did not interact with immunity and inflammation status to influence MetS risk (Supplemental Table S2). According to immunity groups, the KBD, PBD, WSD, and RBD intake scores are presented in Table 4. PBD scores were the lowest, but WSD and RBD scores were highest in the H-WBC+H-CRP group among all groups (Table 4). A KBD (*p* = 0.001; Bonferroni correction *p* = 0.003), a PBD (*p* = 0.015; Bonferroni correction *p* = 0.045), and an RBD (*p* = 0.013; Bonferroni correction *p* = 0.039) had an interaction with immunity to influence MetS risk (Table 5). MetS risk was associated with H-WBC+L-CRP and H-WBC+H-CRP by 1.94- and 1.86-fold in the low intake of a KBD pattern, respectively, compared to L-WBC+L-CRP. However, in its high intake, MetS risk had an association only with the H-WBC+H-CRP group. In a high KBD, MetS incidence was much higher in H-WBC+H-CRP than L-WBC+L-CRP but not in other groups. However, in a low KBD, MetS was lower in L-WBC+L+CRP than the other groups (Figure 2B). In the low intake of a PBD, immunity status was significantly associated by about two-fold with MetS risk, but there was no significant association between MetS and immunity status in its high intake group (Table 5; Figure 2C). In a low RBD intake, MetS had a positive association with H-WBC+L-CRP, and in the high intake, it was positively associated with H-WBC+H-CRP compared to L-WBC+L-CRP (Table 5). In the high intake of an RBD, MetS incidence was highest in H-WBC+H-CRP among the groups (Figure 2D). WSD did not have a significant association with immunity (Table 5).

## 4. Discussion

MetS is associated with overactivated immunity that can underly the meta-inflammation [5,26]. Immune responses are mainly linked to most WBCs, including monocytes (which may differentiate into macrophages), neutrophils, eosinophils, basophils, natural killer cells, and mast cells. Immunity is related to the release of cytokines and activation of the complement system [27]. Less activation of immunity is associated with a lower risk of metabolic syndrome. Although WBC counts and serum CRP concentrations may not be a precise indicator of an individual’s immune status, they can represent immune status in a clinical setting [4,5,10,11]. The present study demonstrated that they could be potentially valuable clinical markers of immunity. The present study also showed that immunity was positively associated with MetS and its components. Among MetS components, serum glucose concentrations and HbA1c concentrations had the strongest association with immunity. Physical activity and PBD intake were negatively associated with immunity, but a WSD had a positive relationship. The results suggest that individuals with abnormally activated immunity may be susceptible to type 2 diabetes and that physical activity and a PBD can suppress overactivated immunity and improve glucose regulation. 

It is difficult to define the immune status with biochemical parameters. Continuous activation of immunity elevates proinflammatory cytokines, which can also kill host cells in addition to bacteria and viruses. Therefore, immunity should be maintained at a moderate level to facilitate a rapid response. However, abnormal immunity is detrimental to the host’s health. Increased insulin resistance is a common feature in the etiology of MetS, and it impairs β-cell function and reduces β-cell mass, causing the development of type 2 diabetes [28,29]. Chronic, low-grade inflammation is mainly characterized by increased serum CRP concentrations and proinflammatory cytokines, such as tumor necrosis factor (TNF)-α, interleukin (IL)-1, and IL-6. WBC counts are also elevated under a chronic low-grade inflammation state and vice versa. WBC counts and serum CRP concentrations are measured in routine check-ups, and they can be representative of immunity status. 

MetS is closely related to chronic, low-grade inflammation indicated by increased serum CRP concentrations [30], as shown in the present study. In a Chinese population, serum CRP concentrations were positively associated with MetS risk by 4.82 (1.89–12.3) but only in women in a five-year follow-up study [31]. However, in the present study, serum CRP concentrations did not associate with genders and were related to MetS less than WBC counts. However, only a few studies have investigated WBC counts as an index of MetS. Some studies have reported that WBC counts are significantly positively associated with body mass index, hypertension, and triglyceride concentrations in type 2 diabetic patients [27]. In the UK Biobank data, lymphocyte counts are positively associated with SBP and DBP [32]. In the present study, participants with H-WBC+L-CRP and H-WBC+H-CRP had positive associations with MetS and its components compared to those with L-WBC+L-CRP. Moreover, the AUC of WBC counts and serum CRP concentrations was 0.637 and 0.671, respectively, in the ROC curve for MetS diagnosis. Thus, high WBC counts and serum CRP concentrations might be weak risk factors for MetS. Although which subpopulation of WBC is associated with MetS risk, neutrophils and lymphocytes, mainly composed of WBC, may be involved in MetS risk in previous studies [32,33,34]. Furthermore, the increased ratio of neutrophils and lymphocytes may be an independent risk factor of MetS. Thus, neutrophils may be more responsible for MetS risk although neutrophils and lymphocytes are also associated with its risk. 

MetS is well known to be modulated by lifestyles, including diets and physical activity [35]. However, no single dietary or physical activity prescription is sufficient to prevent and alleviate MetS [35]. Nutrient intakes, such as a low-fat diet and a low-carbohydrate diet, have been studied to ameliorate MetS, but recently dietary patterns, such as the Mediterranean-style diet and dietary approaches to stop hypertension, have shown promise for reducing MetS [35]. Since systemic inflammation and blood WBC counts are associated with MetS risk, it is crucial to maintain close to the lower limit of WBC, which might be achieved through lifestyle modifications, including diets. The present study showed that energy and fat intakes were not associated with immunity as determined by WBC count and serum CRP concentrations. 

However, three dietary patterns in the PCA analysis had a significant association with immunity in the present study. The PBD had a negative association with H-WBC+L-CRP compared to the L-WBC+L-CRP. These results are consistent with prospective studies in which a PBD including fruits, vegetables, cereals, legumes, nuts, vegetable oils, possibly dairy products, and eggs, similar to the PBD pattern in the present study, reduces the incidence of MetS [36,37]. No studies have reported that a PBD is associated with immunity. However, a PBD is associated with substantially reducing proinflammatory cytokines, including serum CRP, TNF-α, and IL-6 concentrations, in obese participants [38]. The PBD is also reported to be associated with promoting gut microbiome health due to the dietary fiber in plant-based foods [39]. Dietary fiber intakes have been consistently reported to increase lactic acid bacteria, such as Ruminococcus, *Eubacterium rectale*, and *Roseburia*, and reduce Clostridium and Enterococcus species [39]. A PBD promotes the production of short-chain fatty acids (SCFAs) by fermenting dietary fibers and increases gut contents of SCFA, especially propionate and butyrate, to promote an anti-inflammatory state and immunity through the gut-liver-brain axis [12]. As a result, a PBD is associated with a lower risk of MetS [39]. 

Increased immune and inflammatory responses are involved in β2-adrenergic stimulation by activating the hypothalamus-pituitary gland-adrenal gland in obese persons, and physical activity interferes with those responses [40]. Serum glutamate concentrations may play a pivotal role in the division of leucocytes, and physical training decreases serum glutamate concentration (<500 uM) that may influence the decrease of WBC counts to alter immune function [41]. The present study showed that physical activity decreases WBC counts regardless of serum CRP concentrations, consistent with other studies [40,42]. 

This study is novel in its demonstration that overactivated immunity may be associated with MetS risk, primarily type 2 diabetes. This study also demonstrated that a PBD and physical activity might mitigate inflammation and immunity overactivation. It may guide new research directions for the discovery of new therapeutic approaches for predicting and managing MetS. However, this study had some limitations. The data were cross-sectionally collected although they came from a large city hospital cohort study. The results could not be interpreted to establish cause-and-effect relationships. Second, the nutrient and food intake may be underestimated or overestimated since the usual nutrient intake was calculated from SQFFQ data composed of 103 common foods in Korea. However, the SQFFQ was validated by three-day food records, and the SQFFQ data were reliable. Finally, the endpoint of innate immunity was evaluated using WBC counts and serum CRP concentrations that might not fully represent the individual’s innate immune status. However, the index can be applied to measuring chronic, low-grade inflammation by activating innate immunity in a clinical setting.

In conclusion, high serum CRP concentrations and high WBC counts, indicating overactivated immunity, were positively associated with MetS risk, primarily type 2 diabetes. All MetS components had a positive association with immunity, and hyperglycemia had the most significant positive association with the H-WBC and H-CRP group. A PBD and physical activity were negatively associated with abnormal immunity, and WSD had a positive association. Therefore, overactivated immunity may act as a risk factor for MetS, and dietary modification and physical activity might shift abnormal immunity into a lower value of the normal range. Chronic overactivation of immunity provides a new model for the MetS risk, possibly guiding new research directions to discover new therapeutic approaches for predicting and managing MetS risk. Further studies need to examine the association of immunity and inflammation status with cardiovascular diseases, including myocardial infarction and cerebral stroke.

## Figures and Tables

**Table 1:** General characteristics and frequencies of various diseases of the participants according to immunity status.

	L-WBC+L-CRP (*n* = 25,604)	H-WBC+L-CRP (*n* = 13,880)	L-WBC+H-CRP (*n* = 464)	H-WBC+H-CRP (*n* = 820)
Age (years)	53.9 ± 7.8 ^b^	53.2 ± 8.4 ^c^	55.2 ± 7.9 ^a^	54.5±8.2 ^ab^ ***
Gender (Male *N*, Yes %)	7234 (28.3)	6084 (48.8)	168 (36.2)	370 (45.1) ^†††^
Physical activity (*N*, Yes %)	13,726 (56.4)	6895 (51.6)	242 (56.0)	372 (47.6) ^†††^
Smoker (*N*, %)	1541 (6.16)	2666 (19.7)	35 (7.79)	174 (3.96) ^†††^
Metabolic syndrome (*N*, Yes %)	2541(10.4)	2663 (19.8)	83 (19.4)	212 (26.7) ^†††^
Obesity (*N*, %)	6985 (28.4)	5209 (38.6)	168 (39.2)	356 (44.8) ^†††^
Type 2 diabetes (*N*, %)	1722 (7.05)	1879 (14.0)	63 (14.5)	163 (20.8) ^†††^
Hypertension (*N*, %)	5157 (21.1)	3973 (29.7)	133 (30.6)	275 (35.4) ^†††^
Cardiovascular disease (*N*, %)	894 (3.66)	635 (4.74)	20 (4.63)	40 (5.10) ^†††^
Myocardial infarction (*N*, Yes %)	642 (2.63)	457 (3.41)	14 (3.24)	26 (3.32) ^†††^
Stroke (*N*, %)	266 (1.09)	199 (1.48)	6 (1.39)	15 (1.91) ^††^

Immunity was categorized into four groups: L-WBC+L-CRP, H-WBC+L-CRP, L-WBC+H-CRP, and H-WBC+H-CRP. Their cutoffs were <6.2 × 10^9^/mL for white blood cell counts (L-WBC) and <0.5 mg/dL serum CRP concentrations (L-CRP) for low levels. H-WBC and H-CRP represented their high levels. N, Number. *** Significant difference by the immunity groups in one-way ANCOVA test at *p* < 0.001. ^††^ Significant difference among the groups in categorical variables by χ^2^ test at *p* < 0.01. ^†††^ at *p* < 0.001. ^a,b,c^ Different letters indicate significant differences among the groups in the Tukey test at *p* < 0.05.

**Table 2:** Adjusted means and 95% confidence intervals of metabolic syndrome-related parameters according to immunity status.

	L-WBC+L-CRP (*n* = 25,604)	H-WBC+L-CRP (*n* = 13,880)	L-WBC+H-CRP (*n* = 464)	H-WBC+H-CRP (*n* = 820)
WBC (10^9^/L)	4.83 (4.82–4.85) ^c^	7.18 (7.17–7.20) ^b^	4.84 (4.54–4.93) ^c^	7.97 (7.91–8.04) ^a^***^#^
Serum CRP (mg/dL)	0.08 (0.08–0.08) ^d^	0.11 (0.10-0.11) ^c^	1.2 (1.179–1.23) ^b^	1.39 (1.37–1.41) ^a^***^#^
BMI (kg/m^2^)	23.6 (23.6–23.6) ^c^	24.3 (24.2–24.3) ^b^	24.3 (24.1–24.6) ^a^	24.9 (24.7–25.1) ^a^***^#^
Waist circumferences (cm)	80.2 (80.2–80.3) ^b^	80.7 (80.7–80.8) ^a^	80.5 (80.0–80.9) ^ab^	80.9 (80.6–81.2) ^a^***^#^
Serum glucose (mg/dL)	94.2	96.9	97.4	100.2
	(93.9–94.4) ^c^	(96.6–97.3) ^b^	(95.6–99.2) ^b^	(98.9–102) ^a^***^#^
Blood HbA1c (%)	5.64 (5.63–5.65) ^c^	5.82 (5.81–5.83) ^b^	5.77 (5.70–5.83) ^b^	5.96 (5.92–6.01) ^a^***^#^
Serum total cholesterol (mg/dL)	197 (197–198) ^b^	200 (199–200) ^a^	193 (190–196) ^b^	196 (194–199) ^b^***^#^
Serum HDL (mg/dL)	55.2 (55.0–55.3) ^a^	53.5 (53.3–53.8) ^b^	53.1 (52.0–54.3) ^b^	52 (51.1–52.8) ^c^***^#^
Serum LDL (mg/dL)	118 (118–119)	118 (117–119)	116 (113–119)	119 (116–122)
Serum TG (mg/dL)	119 (118–120) ^b^	140 (138–141) ^a^	118 (111–126) ^b^	126 (119–131) ^b^***^#^
SBP (mmHg)	122 (122–122) ^b^	124 (123–124) ^a^	122 (121–123) ^ab^	123 (122–124) ^a^**
DBP (mmHg)	75.1 (75.0–75.2) ^b^	75.8 (75.1–76.4) ^ab^	75.3 (74.5–76.2) ^ab^	75.9 (75.7–76.1) ^a^**
Serum AST	23.4	23.9	24.7	30.7
(IU/L)	(23.1–23.7) ^b^	(23.4–23.9) ^b^	(22.3–27.0) ^b^	(28.9–32.4) ^a^***^#^
Serum ALT	21.4	22.8	23.1	27.8
(IU/L)	(21.1–21.8) ^b^	(22.5–23.2) ^b^	(21.0–25.2) ^b^	(26.3–29.4) ^a^***^#^

Immunity was categorized into four groups: L-WBC+L-CRP, H-WBC+L-CRP, L-WBC+H-CRP, and H-WBC+H-CRP. Their cutoffs were <6.2 × 10^9^/L for white blood cell counts (L-WBC) and <0.5 mg/dL serum CRP concentrations (L-CRP) for low levels. H-WBC and H-CRP represented their high levels. SBP, systolic blood pressure; DBP, diastolic blood pressure; AST, aspartate aminotransferase; ALT, alanine aminotransferase; TG, triglyceride. Adjusted for covariates, including age, gender, body mass index, daily energy intake, income, education, the incidence of arthritis, allergy, asthmas, gastritis, gastric ulcer, gallstone, bronchitis, urethritis, physical activity, alcohol intake, and smoking status. ** Significant difference by the immunity groups in one-way ANCOVA test at *p* < 0.01 and *** at *p* < 0.0001. # Significant difference by the immunity groups in one-way ANCOVA test with Bonferroni correction at *p* < 0.002. ^a,b,c,d^ Different letters indicated significant differences among the groups in the Tukey test at *p* < 0.05.

**Table 3:** Adjusted odds ratio and 95% confidence intervals for metabolic syndrome and various metabolic-related parameters by innate immunity status.

	L-WBC+L-CRP (*n* = 25,604)	H-WBC+L-CRP (*n* = 13,880)	L-WBC+H-CRP (*n* = 464)	H-WBC+H-CRP (*n* = 820)
Age (≥55 years)	1	0.90 (0.86–0.95) ***	1.53 (1.23–1.89) ***	1.21(1.03–1.42) *
Gender (Female)	1	0.74 (0.62–0.87) *	0.09 (0.01–0.70) *	1.09 (0.70–1.70)
Metabolic syndrome (Yes)	1	1.75 (1.64–1.88) ***	1.37 (1.05–1.81)	1.86 (1.54–2.24) **
BMI (≥25 kg/m^2^)	1	1.49 (1.42–1.56) ***	1.57 (1.29–1.91) ***	1.87 (1.62–2.17) ***
Waist circumference (>90 cm for men; >85 cm for women)	1	1.15 (1.07–1.23) *	1.09 (0.81–1.46)	1.03 (0.82–1.29)
Plasma glucose (>126 mg/dL)	1	1.94 (1.80–2.09) ***	1.74 (1.32–2.01) ***	2.6 (2.15–3.14) ***
HbA1c (>7.5% )	1	2.56 (2.20–2.96) ***	2.87 (1.80–4.58) ***	4.72(3.52–6.33) ***
Serum total cholesterol (>240 mg/dL)	1	1.26 (1.19–1.32) ***	0.89 (0.71–1.13)	1 (0.84–1.19)
Serum HDL (40 for men, 50 mg/dL for women)	1	1.3 1.24–1.37) ***	1.37 (1.11–1.69) *	1.69 (1.45–1.97) ***
Serum LDL (>160 mg/dL)	1	1.2 (1.13–1.27) ***	1.04 (0.81–1.35)	1.02 (0.84–1.24)
Serum TG (>200 mg/dL)	1	1.55 (1.45–1.66) ***	1 (0.77–1.30)	0.95 (0.79–1.15)
SBP (>140 mmHg)	1	1.15 (1.09–1.22) ***	0.94(0.75–1.18)	1.18 (1.01–1.38) *
DBP (>90 mmHg)	1	1.30 (1.19–1.43) ***	0.98(0.69–1.41)	1.43 (1.14–1.81) **
Serum ALT (>40 IU)	1	1.38 (1.26–1.50)	1.61 (1.15–2.24)	1.32 (1.03–1.69)
Serum AST (>40 IU)	1	1.24 (1.11–1.39)	1.49 (0.98–2.27)	2.12 (1.63–2.77) ***
Physical activity	1	0.82 (0.78–0.85) ***	0.94 (0.77–1.16)	0.67 (0.57–0.78) ***

Innate immunity was categorized into four groups: L-WBC+L-CRP, H-WBC+L-CRP, L-WBC+H-CRP, and H-WBC+H-CRP. Their cutoffs were <6.2 × 10^9^/mL for white blood cell counts (L-WBC) and <0.5 mg/dL serum CRP concentrations (L-CRP) for low levels. H-WBC and H-CRP represented their high levels. Values represent the adjusted odds ratio and 95% confidence intervals for the participants with the given criteria in the parenthesis after the parameter in the table. Adjusted for covariates, including age, gender, body mass index (BMI), daily energy intake, income, education, the incidence of arthritis, allergy, asthmas, gastritis, gastric ulcer, gallstone, bronchitis, urethritis, physical activity, alcohol intake, and smoking status. L-WBC+L-CRP was a reference for the association analysis. SBP, systolic blood pressure; DBP, diastolic blood pressure; AST, aspartate aminotransferase; ALT, alanine aminotransferase; TG, triglyceride; eGFR, estimated glomerular filtration rate. Adjusted for covariates, including age, gender, body mass index, daily energy intake.* Significantly different from the L-WBC+L-CRP in logistic regression analysis at *p* < 0.05, ** at *p* < 0.01, and *** at *p* < 0.001.

**Table 4:** Daily nutrient intake and dietary pattern scores according to the immunity status.

	L-WBC+L-CRP	H-WBC+L-CRP	L-WBC+H-CRP	H-WBC+H-CRP
	(*n* = 25,604)	(*n* = 13,880)	(*n* = 464)	(*n* = 820)
Energy (% EER)	96.0 ± 0.19	96.1 ± 0.26	92.7 ± 1.41	95.2 ± 1.06
CHO (% En)	71.7 ± 0.04 ^a^	71.4 ± 0.06 ^b^	71.5 ± 0.32 ^ab^	71.5 ± 0.24 ^ab^**
Fat (% En)	13.9 ± 0.03 ^b^	14.2 ± 0.05 ^a^	14.1 ± 0.25 ^ab^	14.0 ± 0.19 ^ab^**
Protein (% En)	13.3 ± 0.02	13.4 ± 0.02	13.4 ± 0.12	13.4 ± 0.09
V-A (μg RE)	476 ±2.13	477 ± 2.92	460 ± 15.6	459 ± 11.7
Carotene (mg)	2362 ± 11.5	2367 ± 15.8	2262 ± 84.1	2280 ± 63.2
V-C (mg)	104 ± 0.41 ^a^	102 ± 0.56 ^b^	98.4 ± 2.99 ^ab^	97.9 ± 2.25 ^ab^*
Fiber (g)	5.65 ± 0.02 ^a^	5.61 ± 0.02 ^b^	5.33 ± 0.13 ^ab^	5.41 ± 0.10 ^ab^*
Coffee (cups/week)	3.7 ± 0.02 ^b^	3.9 ± 0.03 ^a^	3.5 ± 0.14 ^b^	3.9 ± 0.10 ^a^***
Alcohol (g)	17.4 ± 0.31 ^a^	14.9 ± 0.42 ^b^	16.6 ± 2.24 ^a^	15.8 ± 1.69 ^ab^*
KBD (scores)	−0.033 ± 0.006	−0.014 ± 0.008	−0.089 ± 0.044	−0.029 ± 0.034
PBD (scores)	0.022 ± 0.006 ^a^	−0.011 ± 0.008 ^b^	−0.002 ± 0.045 ^ab^	−0.084 ± 0.033 ^c^**
WSD (scores)	0.005 ± 0.006 ^c^	0.043 ± 0.009 ^b^	0.045 ±0.046 ^ab^	0.061 ± 0.035 ^a^*
RBD (scores)	−0.001 ± 0.006 ^b^	0.021 ± 0.009 ^a^	0.068 ± 0.047 ^ab^	0.058 ± 0.035 ^ab^*

Innate immunity was categorized into four groups: L-WBC+L-CRP, H-WBC+L-CRP, L-WBC+H-CRP, and H-WBC+H-CRP. Their cutoffs were < 6.2 × 10^9^/mL for white blood cell counts (L-WBC) and <0.5 mg/dL serum CRP concentrations (L-CRP) for low levels. The high levels of white blood cell (WBC) counts and serum CRP concentrations were represented as H-WBC and H-CRP, respectively. Adjusted for age, gender, body mass index, daily energy intake, income, education, the incidence of arthritis, allergy, asthmas, gastritis, gastric ulcer, gallstone, bronchitis, urethritis, physical activity, alcohol intake, and smoking status. EER, estimated energy requirement; CHO, carbohydrates; V-A, vitamin A; V-C, vitamin C; KBD, Korean balanced diet; PBD, plant-based diet; WSD, Western-style diet; RBD, rice-based diet. Values represented adjusted means and standard errors. * Significant difference by the immunity groups in one-way ANCOVA test at *p* < 0.05, ** at *p* < 0.01, *** at *p* < 0.001. ^a,b^ Different letters indicated significant differences among the groups in the Tukey test at *p* < 0.05.

**Table 5:** Innate immunity and diet/lifestyle interaction and adjusted odds ratio of metabolic syndrome-related parameters in the low- and high-intake of diet patterns after covariate adjustments.

	L-WBC+L-CRP (*n* = 25,604)	H-WBC+L-CRP (*n* = 13,880)	L-WBC+H-CRP (*n* = 464)	H-WBC+H-CRP (*n* = 820)	Immunity, Lifestyle, and Diet Interaction
Low physical activity	1	1.805 (1.637–1.990) *	1.628 (1.105–2.398)	2.057 (1.582–2.675) *	0.650 ^1^
High physical activity	1	1.835 (1.677–2.008) **	1.396 (0.955–2.039)	2.047 (1.561–2.684) **	
Non-smokers	1	1.86	1.59	1.64	0.042
		(1.71–2.02) ***	(1.16–2.16)	(1.28–2.12)	0.081 ^2^
Smokers	1	1.71	1.45	2.35	
		(1.52–1.91)	(0.90–2.35)	(1.77–3.11) ***	
Low intake of a KBD	1	1.94	1.49	1.86	0.001
		(1.79–2.11) ***	(1.08–2.05)	(1.47–2.34) *	0.003
High intake of a KBD	1	1.39	1.11	1.84	
		(1.23–1.57)	(0.65–1.92)	(1.31–2.57) *	
Low intake of plant-based diet	1	1.79	1.25	2.04	0.015
		(1.66–1.94) ***	(0.89–1.75)	(1.65–2.52) ***	0.045
High intake of a plant-based diet	1	1.48	1.66	1.03	
		(1.07–1.92)	(1.04–2.65)	(0.67–1.59)	
Low intake of	1	1.77	1.59	1.53	0.013
rice-based diet		(1.63–1.91) ***	(1.16–2.18)	(1.22–1.94)	0.039
High intake of	1	1.95	1.53	3.13	
rice-based diet		(1.73–2.21)	(0.95–2.44)	(2.29–4.29) ***	

Innate immunity was categorized into four groups: L-WBC+L-CRP, H-WBC+L-CRP, L-WBC+H-CRP, and H-WBC+H-CRP. Their cutoff points were <6.2 × 10^9^/L for white blood cell counts (L-WBC) and <0.5 mg/dL serum CRP concentrations (L-CRP) for low levels. The high levels of white blood cell (WBC) counts and serum CRP concentrations were represented as H-WBC and H-CRP, respectively. KBD, Korean balanced diet. The cutoff points for dietary patterns were the 70th percentiles. Values represent the adjusted odds ratio and 95% confidence intervals. ^1^, *p*-value for the interaction of immunity categories and nutrient and dietary patterns influencing metabolic syndrome risk using two-way ANCOVA with main effects (innate immunity and nutrients) and their interaction term after adjusting for covariates. ^2^, *p*-value for the interaction using two-way ANCOVA with the Bonferroni correction. Adjusted for age, gender, body mass index, daily energy intake, income, education, the incidence of arthritis, allergy, asthmas, gastritis, gastric ulcer, gallstone, bronchitis, urethritis, physical activity, alcohol intake, and smoking status. * Significantly different from the L-WBC+L-CRP in logistic regression at *p* < 0.05 ** at *p* < 0.01, and *** at *p* < 0.001.

## Data Availability

The raw data involved in this study will be available by the authors to any qualified researcher.

## Supplementary Materials

The following are available online at https://www.mdpi.com/article/10.3390/nu13072308/s1, Table S1: General characteristics and incidence of various diseases of the participants according to immunity status and genders; Table S2: Adjusted odds ratio (ORs) and 95% confidence intervals (CIs) of high WBC or serum CRP concentrations for metabolic syndrome and various metabolic-related parameters; Table S3: Factor loadings of food groups in dietary patterns identified using principal component analysis

## Abbreviations

MetS: metabolic syndrome; WBC, white blood cells; CRP, serum C-reactive protein concentrations; L-WBC, <6.2 × 10^9^/L WBC; L-CRP, <0.5 mg/dL serum CRP concentrations; H-WBC, ≥6.2 × 10^9^/L WBC; H-CRP, ≥0.5 mg/dL serum CRP concentrations; KoGES, Korean Genome and Epidemiology Study; SQFFQ, semi-quantitative food frequency questionnaire; ORs, odds ratios; CI, 95% confidence intervals; SCFAs, short-chain fatty acids; TNF, tumor necrosis factor; IL, interleukin; ROC, receiver operating characteristic.
