# Supplementary material for: A Positive Association of Overactivated Immunity with Metabolic Syndrome Risk and Mitigation of Its Association by a Plant-Based Diet and Physical Activity in a Large Cohort Study
Source: Nutrients. 2021 Jul 5;13(7):2308. doi: 10.3390/nu13072308 (PMC8308429; doi:10.3390/nu13072308)
Supplement: Supplementary file 1 [file nutrients-13-02308-s001.zip › nutrients-1276598-supplementary.pdf]

Table S1. General characteristics and incidence of various diseases of the participants according to innate immunity status and genders

	Men				Women			
	L-WBC+L-CRP (n=7,234)	H-WBC+L-CRP (n=6,084)	L-WBC+H-CRP (n=168)	H-WBC+H-CRP (n=370)	L-WBC+L-CRP (n=17,450)	H-WBC+L-CRP (n=7,494)	L-WBC+H-CRP (n=277)	H-WBC+H-CRP (n=428)
Age (years)	55.1±8.3[1a]	54.5±8.5[b]	55.6±8.2[ab]	55.5±8.4[ab***]	53.1±7.6[b]	52.4±8.1[c]	54.5±7.8[a]	53.6±7.9[b***]
Physical activity (N, Yes %)	4,368 (62.8)	3,241(54.9)	101(64.7)	185 (52.0)[***]	9,358(53.8)	3,654 (49.0)	141 (51.1)	187 (43.9)[***]
Smoker (N, %)	1,307 (18.8)	2,385 (40.4)	34 (21.8)	1,51 (42.4)[***]	234 (1.35)	281 (3.77)	1 (0.36)	23 (5.39)[***]
Metabolic syndrome (N, Yes %)	930(13.3)	1311 (22.1)	29 (18.5)	105 (29.4)[***]	1,611 (9.2)	1,352 (18.0)	54 (19.5)	107 (25.0)[***]
Obesity (N, %)	2,634(37.7)	2,552 (43.0)	66 (42.0)	154 (43.1)[***]	4,351 (24.9)	2,657 (35.5)	102 (36.8)	202 (47.2)[***]
Hypertension (N, %)	1,967(28.2)	2,011(33.9)	53 (33.8)	136(38.1)[***]	3,190 (18.3)	1,962 (26.2)	80 (28.9)	139 (32.5)[***]
Type 2 Diabetes (N, %)	769 (11.0)	963 (16.2)	23 (14.7)	79 (22.1)[***]	953 (5.46)	916 (12.2)	40 (14.4)	84 (19.6)[***]
Cardiovascular disease (N, %)	421 (5.93)	390 (6.58)	7 (4.49)	25 (6.74)	487 (2.75)	245 (3.27)	13 (4.71)	16 (3.74)[*]
Myocardial infarction (N, Yes %)	290 (4.15)	278 (4.69)	5 (3.21)	15 (4.21)	352 (2.02)	179 (2.39)	9 (3.26)	11 (2.57)
Stroke (N, %)	131 (1.10)	122 (2.06)	2 (1.28)	10 (2.81)	135 (0.77)	77 (1.03)	4 (1.45)	5 (1.17)

[1]Means ± standard deviations. Immunity was categorized into 4 groups: L-WBC+L-CRP, H-WBC+L-CRP, L-WBC+H-CRP, and H-WBC+H-

CRP. Their cutoffs were < 6.0 X10^9/ml for white blood cell counts (L-WBC) and < 0.5 mg/dL serum CRP concentrations (L-CRP) for low levels. Their high levels were represented by H-WBC and H-CRP. N, Number.

[***] Significant difference by the immunity groups in one-way ANCOVA test at P<0.0001.

[***] Significant difference among the groups in categorical variables by X^2 test at P<0.0001.

[a,b,c] Different letters indicated significant differences among the groups in the Tukey test at P<0.05.

Supplementary Table S2. Adjusted odds ratio (ORs) and 95% confidence intervals (CIs) of high WBC or serum CRP concentrations for metabolic syndrome and various metabolic-related parameters

	High WBC (≥6.2X10; n=11,363)	High CRP (≥0.5 mg/dL; n=2,018)
Age (<55 years)[1]	1.105 (1.012-1.206) *	1.240 (1.036-1.483) *
Gender (Female)	1.051 (0.895-1.233)	0.807 (0.566-1.152)
MetS (no MetS)	1.490 (1.349-1.646) ***	1.465 (1.232-1.742) ***
BMI (<25 kg/m^2)	1.188 (1.095-1.290)***	1.222 (1.036-1.442) *
Waist circumferences (M: <90 cm; F: <85 cm)	1.256 (1.148-1.374) ***	1.465 (1.232-1.742) ***
Serum glucose (<126 mg/dL)	1.232 (1.132-1.341) ***	1.110 (0.936-1.316)
Blood HbA1c (<6.5%)	1.934 (1.715-2.181) ***	1.128 (0.890-1.430)
Serum total cholesterol (< 230 mg/dL)	1.528 (1.387-1.684) ***	0.868 (0.703-1.071)
Serum HDL (M: ≥40 F: ≥ 50 mg/dL)	1.107 (1.006-1.220) *	1.548 (1.288-1.861)***
Serum LDL (<160 mg/dL)	1.383 (1.231-1.553) ***	1.016 (0.799-1.291)
Serum TG (<150 mg/dL)	1.536 (1.413-1.671) ***	0.920 (0.778-1.088)
SBP (<130 mmHg)	1.113 (1.024- 1.210) *	0.954 (0.804-1.132)
DBP (<90 mmHg)	1.044 (0.922-1.181)	1.005 (0.783-1.289)
Serum AST (<35 IU/L)	1.150 (1.005-1.316) *	1.599 (1.263-2.024) ***
Serum ALT (<35 IU/L)	1.210 (1.088-1.346) **	0.993 (0.794-1.240)

WBC, white blood cell counts; CRP, serum C-reactive protein concentrations

[1] ( ) reference for ORs

[2] Adjusted ORs and 95% confidence intervals after age, gender, body mass index (BMI), residence area, education level, income, daily energy intake, physical activity, smoking and alcohol intake,

Supplemental Table S3. Factor loadings of food groups in dietary patterns identified using principle component analysis

	Korean balanced diet		Plant-based diet		Western-style diet		Rice-based diet	
Rice	-2		-7		4		93	*
Grains	9		-4		-2		-93	*
Noodles	2		3		62	*	2	
Breads	-7		34		53	*	-5	
Cakes	-8		32		30		6	
Cookies	31		48	*	3		2	
Beans	24		50	*	6		-3	
Tubers	50	*	2		-1		-2	
Kimchi	8		44	*	15		5	
Eggs	-4		15		75	*	-4	
Fast foods	67	*	39		-2		-1	
Green vegetables	70	*	29		1		2	
White vegetables	74	*	29		-4		3	
Mushroom	49	*	40	*	-6		-4	
Fatty fishes	53	*	22		12		0	
White fishes	66	*	17		13		0	
Crabs	49	*	3		19		1	
Processed meats	18		15		6		-1	
Red meats	46	*	-7		41	*	8	
Soups	16		4		65	*	-4	
Chicken	33		-5		39	*	4	
Seaweed	44	*	41	*	-2		-4	
Milk and its products	11		49	*	2		0	
Beverages	19		32		6		2	
Coffee	10		0		18		15	
Teas	14		-8		24		14	
Fruits	19		47	*	-6		-6	
Korean fermented foods	50	*	0		5		2	
Alcohols	18		-28		16		6	
Nuts	-2		50	*	6		-5	
Variance Explained by Each Factor	3.533		2.493		2.296		1.798	

Printed values are multiplied by 100 and rounded to the nearest integer.

Values greater than 0.40 are flagged by an '*'.
